# Work-life integration among nurse educators: a meta-synthesis

**DOI:** 10.3389/fgwh.2024.1287484

**Published:** 2024-05-30

**Authors:** Sonja Erasmus, Charlene Downing, Nompumelelo Ntshingila

**Affiliations:** Department of Nursing, Faculty of Health Sciences, University of Johannesburg, Johannesburg, South Africa

**Keywords:** work-life balance, nurse educator, systematic review, faculty (MeSH), nurse academic

## Abstract

**Background:**

Work-life integration has been extensively researched in various contexts. Women dominate the nursing profession, but work-life integration is essential for men and women since both are expected to focus equally on their families and careers. The nursing faculty perceives nurse educators’ work environment as undervalued, lacking support, and limited time to grow and carry the heavy workload.

**Method:**

A qualitative meta-synthesis of studies between 2013 and 2023 was conducted using ScienceDirect, EBSCO Host, Sage and Sabinet databases. Seven articles related to the research phenomenon were retrieved.

**Conclusion:**

The resulting themes revolved around two central aspects: nurse educators’ work and life integration. Nurse educators face various challenges with work-life integration and often view their failure as a personal rather than a societal issue. However, as much as achieving work-life integration is personal, there is a call for employers in academic environments to improve workplace policies, like better-paid maternity leave, affordable quality childcare, and social support. Furthermore, nurse educators’ line managers should display warmth and encouragement about personal challenges affecting nurse educators.

## Introduction

Meta-synthesis is an increasingly popular means of synthesising qualitative research results to achieve findings of greater scope, generalisability, conceptual development, or practical utility than can be achieved in any single primary study (1). Qualitative meta-syntheses are essential for making sense of multiple research studies and have the potential to identify gaps and omissions in each research group. The approach can also provide deeper dimensions and interpretations of qualitative studies (2). A meta-synthesis method aims to capture the increasing volume of qualitative research, facilitate knowledge transfer, and bring a wide range of participants and descriptions together (3, 4). While the number of qualitative research studies on nurse educators’ work-life integration has increased over the past decade, little is known about how a collective body of qualitative research contributes to our understanding of this topic within this field of research. This study integrates/synthesises evidence from qualitative studies of nurse educators’ work-life integration.

Frone's (5) fourfold taxonomy characterises work-life integration as reducing conflict and increasing work-home enrichment. The concept of work-life integration was first used in the United Kingdom in the 1970s, (6) when company policies and regulations allowed employees to work effectively and efficiently and provided flexible time to work through personal difficulties. Rawal (7) refers to work-life integration as maintaining an ideal balance between work (career and work goals) and personal life (fitness, enjoyment, relaxation, spiritual aspirations and family). Work-life integration thus involves an equilibrium between work and private life, both of which bring satisfaction to the individual (8). Work-life integration is ultimately important because it drives employees’ attitudes and behaviours, such as job performance, job satisfaction, and organisational citizenship behaviour (9, 10). Wolor, Kurnianti, Zahra and Martono (11) concur that work-life integration has a vital impact on employee productivity and performance. It is a topical issue of increasing concern due to globalisation, the intrusion of new technologies into personal life, overlapping work and family time, new organisational systems, and changes like work (12).

As stated, work-life integration has been extensively researched in various contexts (13–15). Women dominate the nursing profession, yet work-life integration is important for all genders as these are expected to focus equally on their families and careers (16). Work-life integration must also be extended to men and women working within the nursing education institution. (17). Nurse educators must meet student needs and balance the ongoing demands of high professional expectations, which include clinical competencies, regulatory bodies, and outcomes related to teaching, service, scholarship, and practice (18) Overall, nursing education around the world continues to suffer from underinvestment, static and rigid curricula, a lack of interprofessional preparation of nurses, and a lack of coordinated collaboration and support from stakeholders (19).

Nurse educators perform several duties or functions in their workplace. One of these functions is classroom teaching, where nurse educators spend significant time preparing lectures. Another function is the care given to nursing students in their community and the associated learning experiences in the hospital. Nurse educators must also review individual student results (e.g., exam papers, case studies, etc.,) (20). In some educational contexts, nurse educators have the added role of supervising research and promoting publications. Because of this multitasking, nurse educators are often under pressure and struggle to integrate work and family responsibilities; nurse educators in academia have readily acknowledged a lack of work-life integration (21, 22). This imbalance has negative emotional and professional consequences, such as increased anxiety, depression, burnout, and decreased productivity (21).

Despite significant research on work-life integration, there has been no improvement in this area. The COVID-19 pandemic also worsened the challenges of work-life integration among nurse educators (23). Also, according to Lakkoju and Jeyalakshmi, (24) there will never be one work-life integration method that fits everyone because everyone has different views of life and priorities; therefore, individuals’ work-life integration ideals differ.

The nursing faculty perceives nurse educators’ current work environment as undervalued, lacking support, and with limited time to grow and carry the heavy workload (25). The ideal work environment, as perceived by nurse educators, is one where educators feel valued, can engage in open dialogue, understand situations and share person-centred decision-making. Based on this discussion, the paper's purpose was to compile, review and interpret published qualitative studies on nurse educators’ work-life integration and determine how this population can achieve integration.

## Methods

### Design

A meta-synthesis of the selected qualitative studies was conducted. This strategy is defined as the systematic compilation and integration of qualitative research results to expand the understanding of work-life integration and develop a unique interpretation of the research findings (26). In this meta-synthesis, Noblit and Hare's (27) seven meta-ethnography steps were used. Meta-ethnography is an inductive, interpretive approach on which most interpretive qualitative synthesis methods are based (28) and the most used qualitative synthesis approach in health research (29).

### Search methods

The term “nurse faculty” was initially excluded from the search strings when searching for articles related to work-life integration among nurse educators. This exclusion was based on its limited use in South Africa; it is more commonly employed in the United States of America, where the term is prevalent in the context of nurse educators’ working environment within a “nurse/nursing faculty”. After careful consultation and discussion with the research team, the term “nurse faculty” was decided to be included in the search strings to ensure a comprehensive exploration of the topic. Boolean searches offered an effective way to combine keywords with operators such as “and”, “or”, and “not” to generate more relevant results and enhance the search process. Using the “and” operator, the authors ensured that all search results contained items including all the specified terms, thus refining the search output.

The following search strings and keywords were used during the search for articles on work-life integration among nurse educators from four different databases:

• *“Worklife and nurse educators”*
• *“Work-life and nurse educators”*
• *“Work-life and nurse faculty”*
• *“Work-life nurse teacher”*
• *“Worklife nurse teacher”*
• *“Work-life nurse faculty”*
• *“Worklife nurse faculty”*
• *“Work and life nurse educators”*
• *“Work home interference nurse academics”*
• *“Work-life and nurse educators”*
• *“Nurse and an educator work-life”*
• *“Nurse and an educator worklife”*
• *“Working life nurse education”*
• *“Working life nurse academics”*

By including the term “nurse faculty” and using Boolean search logic, the authors aimed to ensure a comprehensive and contextually relevant exploration of work-life integration among nurse educators. The diverse search strings allowed for an extended search of relevant articles, capturing various aspects of the topic. The careful consideration of search strings and keywords contributed to successfully retrieving articles addressing the research topic. This approach ensured that the research findings were tailored to the unique working environment of nurse educators, especially those within a “nurse/nursing faculty” context, facilitating a deeper understanding of their challenges and strategies for achieving work-life integration in this specific professional setting. Databases for the searches included Science Direct, EBSCO Host, SAGE and Sabinet. The dates for the searches ranged from January 2013 until August 2023. The reference lists of the final articles were also screened for quality appraisal and to obtain additional studies that could be included in the meta-synthesis.

### Inclusion and exclusion criteria

This meta-synthesis included qualitative research articles published in English between 2013 and 2023 in credible journals and peer-reviewed. All the articles needed the following terms in their titles: nurse educator/nurse academic/nurse teacher, nurse faculty/work-life/work/working life/work and life/work and home, balance/integration and interference. A prerequisite for the articles was that they had to be from a primary source to ensure the retrieval and use of the correct primary information. The exclusion criteria were all sources that did not relate to nurse educators, as the purpose of the study was to explore nurse educators’ work-life integration.

### Screening

**Figure 1:**
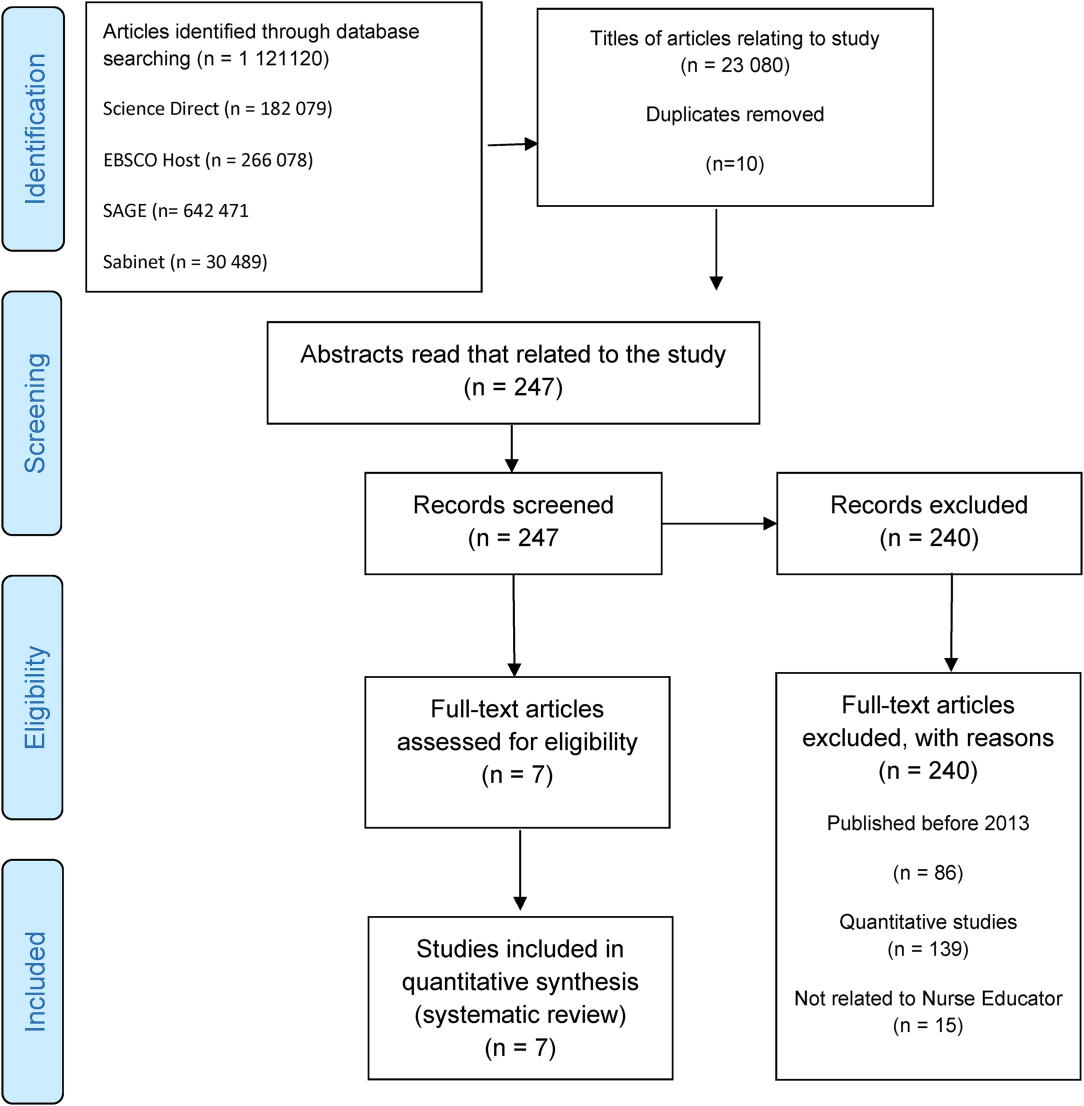
Search strategy and selection of articles.

The screening for this meta-synthesis was conducted manually using Excel spreadsheets at the different screening phases. The first author conducted the initial screening, and then the second and third authors independently verified these by screening them against the set inclusion criteria. Any conflict about the decision to include or exclude an article was resolved through discussion and an external independent coder. A representation of the entire screening process is depicted in the PRISMA Figure 1.

### Quality appraisal

The STARLITE evaluation instrument (30) was used to appraise the articles included in this study. The mnemonic STARLITE (sampling strategy, type of study, approaches, range of years, limits, inclusion and exclusions, terms used, electronic sources) conveys the essential elements for reporting literature searches (30). The three authors independently used the STARLITE tool and measured each article under the criteria of Compliant (2), Partially compliant (1) and Non-compliant (0). The evaluation was conducted using a rating scale: if the article fully met the criteria stipulated, two marks were allocated; if the criteria were partially met, one mark was allocated; and when the criteria were not met, a zero mark was allocated. A percentage was awarded for each article.

### Data abstraction

The three authors read and localised the included articles according to Noblit and Hare's (27) the third step. This was done to understand the individual studies’ themes and metaphors. Each author's themes and metaphors were combined and reported in a single document.

### Synthesis

The authors read and re-read the articles before the data extraction and synthetisation (31). An independent coder participated with the authors in coding the data and identifying the metaphors and themes (32). The study's credibility was ultimately enhanced by gaining input from the independent coder. Direct quotations supporting the themes were then included in the report on the themes of the nurse educators’ work-life integration and how work-life balance can be achieved. Relationships between the results of each study were also established, as indicated in step 4 by Noblit and Hare (27). Thematic analysis was employed to analyse the themes and metaphors across the articles. Themes translating differences and similarities were clustered, and the findings were synthesised.

## Results

### Study characteristics

Articles selected for this study span from January/February 2014 to January 2023, encompassing various scholarly contributions. Among the selected articles, one was published in 2014, another in January 2015, and two in 2016. Subsequently 2019, two additional articles emerged, delving into the research phenomenon, followed by a solitary publication in January 2023. The methodological diversity within these articles is striking, reflecting a multifaceted approach to inquiry. One article adopted a phenomenological lens, delving into the lived experiences of nurse educators, while another embraced a hermeneutic perspective, unravelling the interpretive dimensions of their narratives. Methodological frameworks varied further, with studies employing descriptive methodologies, appreciative inquiry, grounded theory, and storytelling approaches. Additionally, one study pursued an exploratory trajectory, probing novel avenues within the realm of work-life integration among nurse educators.

Geographically, the studies exhibited a notable distribution, with three conducted in Australia and four spanning different states. This geographic diversity enriches the study's scope, capturing nuances and perspectives that reflect varied socio-cultural contexts. Comprehensive details regarding the characteristics of these studies are meticulously presented in Table 1.

**Table 1:** Author and article information.

Article number	Title of the study	Authors	Journal name	Journal impact factor	Date of publication	Data-base
1	The meaning of being a nurse educator and nurse educators’ attraction to academia: A phenomenological study	Laurencelle, F.L	Nurse Education Today	2.533	January 2016	Sciencedirect
		Scanlan, J.M				
		Brett, A.L				
2	Transition from novice adjunct to experienced associate degree nurse educator: A comparative qualitative approach	Paul, P.A	Teaching and learning in Nursing	0.29	January 2015	Sciencedirect
3	Retaining the wisdom: Academic nurse leaders’ reflections on extending the working life of aging nurse faculty	Falk, N.L	Journal of professional nursing	2.045	January/February 2014	Sciencedirect
4	Exploring the experiences of early career academic nurses as they shape their career journey: A qualitative study	Wyllie, A	Nurse Education Today	2.533	January 2019	Sciencedirect
		Levett-Jones, T				
		DiGiacomo, M				
		Davidson, P				
5	Developing resilience: Stories from novice nurse academics	McDermid, F	Nurse Education Today	3.9	January 2016	Sciencedirect
		Peters, K				
		Daly, J				
		Jackson, D				
6	Motivators for nurse educators to persist in their profession: A phenomenological research study	Tufano, V.C	Nursing Education Today	3.9	January 2023	Science Direct
		Summers, E.J.				
		Covington, B.				
7	Work experiences of Nurse Academics: A qualitative study	Singh, C.	Nursing Education Today	3.9	April 2019	Science Direct
		Jackson, D.				
		Munro, I.				
		Cross, W.				

### Quality appraisal

The quality of the articles ranged from 25% to 81%. An interrater agreement and consensus were reached between the three authors after discussion. Table 2 shows the STARLITE scores for each of the articles.

**Table 2:** Starlite evaluation.

Article		S: Sampling strategy (Comprehensive, selective, purposive)	T: Type of studies (Fully reported/partially reported)	A: Approaches (other than electronic searches)	R: Range of years (start date–end date) fully reported/partially reported	L: Limits (English/human)	I: Inclusion and exclusions	T: Terms used	E: Electronic sources	Total	Percentage
1	The meaning of being a nurse educator and nurse educators’ attraction to academia: A phenomenological study	2	2	1	1	2	1	0	0	9	56,25%
2	Transition from novice adjunct to experienced associate degree nurse educator: A comparative qualitative approach	2	2	1	2	2	2	1	0	12	75%
3	Retaining the wisdom: Academic nurse leaders’ reflections on extending the working life of aging nurse faculty	2	2	1	2	2	2	0	2	13	81.25%
4	Exploring the experiences of early career academic nurses as they shape their career journey: A qualitative study	2	2	1	1	2	2	1	0	11	68.75%
5	Developing resilience: Stories from novice nurse academics	2	2	0	2	2	2	0	1	11	68.75%
6	Motivators for nurse educators to persist in their profession: A phenomenological research study	2	2	1	2	2	2	2	2	13	81%
7	Work experiences of Nurse Academics: A qualitative study	2	2	1	2	2	2	2	2	13	81%

Compliant: 2. Partial compliant: 1. Non-compliant: 0.

### Data abstraction

Data, including author details, year of publication, country, research design and methods, and characteristics of participants, were extracted by all the authors independently into Table 3.

**Table 3:** Characteristics of the primary studies included in the study.

Article number	Methodological orientation	Context, city and continent	Sample, sampling method (sm) and sample size (sz)	Themes	Sub-themes	Data collection method	Study limitations
1	Hermeneutic Phenomenological	College or University in a Western Canadian city located in the Western hemisphere.	Sample: nurse educators SM: purposive sampling SZ: 15 females ranging between 36 and 60 years of age (5 with doctoral degree and 10 with masters degrees).	The meaning of being a nurse educator	1. Opportunities 2. Wanting to teach 3. Seeing students learn 4. Contributing to the profession 5. The unattractive 6. Salary 7. Flexibility	The researcher is the data abstraction instrument, retrieved from face-to-face semi-structured interviews with interview guides, observations and documents to find the meaning of the lived experiences.	All participants in the study were female; the perspective of male academics is missing and may differ from female colleagues. There was no distinction between participants with master's and PhD degrees, nor the educational setting. The small sample size from a specific geographic region limits the transferability of the findings.
2	Descriptive	Several campus sites in Pennsylvania located in the Northern hemisphere.	Sample: novice nursing adjuncts and experienced nursing full-time faculty members SM: purposive sampling SZ: 13 novice adjunct female volunteers One novice adjunct male volunteer Seven experienced full-time nursing female faculty members.	1. Knowing requirements: Must read/must follow 2. Evolving teaching role identity 3. Teaching role management 4. Faculty relationship development	No sub-themes	Emails send and individual interviews were recorded by the researcher.	There was limited diversity because all participants were Caucasian females, except for one Caucasian male adjunct.
3	Grounded theory and constant comparative analysis	Large, small, public, private, urban and rural institutions in nine states of America in the Northern hemisphere.	Sample: theoretical sampling SM: academic nurse leaders SZ: nine females (7 Caucasians, 1 African American, and 1 Hispanic).	1. Valuing aging nurse faculty 2. Enduring environmental challenges 3. Recognizing stakeholder incongruence 4. Readjusting	No sub-themes	Audiotaped in person and telephone interviews using an interview guide.	Sample size was limited to nine academic nurse leaders. The study represents only academic nurse leaders’ reflections.
4	A descriptive study that used an appreciative inquiry approach	One sizeable Metropolitan University in Sydney, Australia in the Southern hemisphere.	Sample: early career academic nurses SM: purposive sampling SZ: 11 (2 males and nine females).	1. Embarking on the journey 2. The toil of the journey 3. Fellow travellers on the journey 4. Strategies for a successful journey	No sub-themes	In-depth, semi-structured interviews were recorded, followed by observations and reflections by the researcher.	The findings of this study were limited to one faculty with nurse academics.
5	Storytelling approach	Context: two major universities are in Australia, in the Southern hemisphere, and one is in the United Kingdom, in the Northern hemisphere.	Sample: new nurse academics SM: purposive and snowball sampling SZ:13 women and one man.	1. Developing supportive collegial relationships 2. Embracing positivity 3. Reflections and transformative growth	No sub-themes	Inductive approach. Semi-structured conversational style face to face audio recorded interviews.	No reference made to limitations in the study.
6	Phenomenological qualitative study	In the Midwestern and one in the Southwestern United States.	Nurse educators (*n* = 16) who were teaching in nursing academic settings.	1. Intrinsic motivator aspects 2. Extrinsic motivator aspects	1.1 Love of teaching 1.2 Desire to learn more. 1.3 Satisfaction from service to the profession 1.4 Seeking challenges • Flexibility • Professional advancement/opportunity	Semi-structured interviews, and verbatim transcription	No reference was made to the limitations of the study.
7	Qualitative exploratory design	All states and territories of Australia	Purposive sample of nurse academics (*n* = 19)	(a). Helping students achieve finding satisfaction through student engagement, (b). Working with challenging students, (c). Increased workloads, lack of support and resources, and (d). Difficulty with the retention of newly appointed staff.	No sub-themes	Semi-structured and face-to-face interviews	No reference was made to the limitations of the study.

### Synthesis of participant quotes

The study explored seven scholarly articles thoroughly, meticulously dissecting their insights to glean a nuanced understanding of work-life integration within the domain of nurse education. Employing a systematic thematic analysis, the authors meticulously unearthed key themes and subthemes, illuminating the multifaceted nature of this phenomenon.

Central to the findings were two overarching dimensions: nurse educators’ work integration and personal lives. Within the realm of work integration, the study uncovered a spectrum of experiences, from the allure of conducive teaching environments to the intricacies of professional relationships. These findings were further nuanced through the delineation of subthemes, which underscored the dichotomy between favourable and challenging aspects. Nurse educators celebrated the enriching environments conducive to teaching and learning alongside the camaraderie forged among peers. Conversely, they grappled with role ambiguity, workplace conflict, and the nuanced task of evaluating student performance.

In parallel, the study delved into the personal lives of nurse educators, shedding light on the delicate interplay between parenthood, academic pursuits, and emotional well-being. Here, the discourse reflected the compatibility of familial roles with the demands of academia, the significance of environmental comfort, and the myriad emotions underpinning the academic journey.

A poignant metaphor emerged from the analysis, likening the pursuit of work-life equilibrium to the art of riding a bicycle. Much like the harmonious synchronization of its various components propels a bicycle forward, successful work-life integration hinges on the delicate balance and alignment of the diverse facets of a nurse educator's life. This metaphor encapsulates the essence of the study's findings, underscoring the imperative of holistic integration in navigating the complexities of professional and personal spheres.

### Description of the findings: themes and sub-themes

The research data were thoroughly analysed to identify specific factors related to work-life integration among nurse educators. The findings were grouped into themes, subthemes, and categories, allowing for a structured presentation of the results. Two predominant themes emerged from the analysis: work integration of nurse educators and life integration of nurse educators. Table 4 summarises the themes and sub-themes related to this population's work-life integration.

**Table 4:** A summary of themes and sub-themes of work-life integration of nurse educators.

Themes	Sub-themes
Theme 1: Work Integration of Nurse Educators	Subtheme 1.1: The Attractive Work Environment of Teaching and Learning in Nursing Education
	Subtheme 1.2: Attractive Relationship Journey of Nurse Educators
	Subtheme 1.3: Unattractive Interpersonal Behaviour of Relating to Nurse Educators in the Workplace
	Subtheme 1.4: The Nurse Educators’ Unattractive External Environment
Theme 2: Life Integration of Nurse Educators	Subtheme 2.1: The Attractive Aligning Parenthood and Academia
	Subtheme 2.2: The Ultimate Costs of downplaying parenthood in Academia

### Theme 1: Work integration of nurse educators

This theme explored the physical work environment and professional relationships nurse educators encountered. It encompassed both attractive and unattractive elements.

#### Subtheme 1.1: The Attractive Work Environment of Teaching and Learning in Nursing Education

This subtheme highlighted nurse educators’ commitment to preparing students for success and their role in self-discovery and fulfilling learning needs. Quotes from the articles supported the significance of teaching as a means of contributing to the nursing profession and positively impacting patient care.

*“The comfort of knowing the operation of the clinical affiliation and familiarity of the organization and campus setting increased personal security in the teaching role.”* (33)

*“I do get a feeling of reward knowing that I'm involved with preparing graduates who are educated, skillful, ethical”*. (34)

*“What motivated me was a couple of things. First and fore most my initial career goals were to be either a nurse or a teacher. Knowing that I couldn't do both or that it wasn't really practical, I felt that maybe this might be a good way to marry the two professions. And I've always wanted to, I kind of thought that it would be really neat to be teaching nurses and kind of marry those two professions in that way.” (NE 7)* (35)

#### Subtheme 1.2: The Attractive Relationship Journey among Nurse Educators

Nurse educators’ relationship journey was romanticised and seen as attractive, with strategies for success, reflections leading to transformative growth, and narratives of career-oriented academics. The ability to adapt, persevere, and belong to a research team were emphasised as key strategies for a successful journey.

*“One of the strategies viewed by the participants as key to academic career development was adaptability and the ability to “bend and adjust”. This was explained in several different ways, for example, one of the participants said: “you’ve got to be really flexible”, “be willing to take risks and be wrong” and “to keep reinventing things or to be on that continual cycle of regeneration, improvement””* (36)

*“All adjuncts verbalized the importance of preparation for class/laboratory/clinical and their love to teach.”* (33)

“*I look back and I remember being terrified. I got sent the class outline a couple of days before…. I stood in front of this classroom of students thinking ‘I can't do this; I'm a nurse not a teacher! ….but you know as nurses we tend to just roll with the punches and we just sort of go in and do it ….and I did*.” (37)

#### Subtheme 1.3: Unattractive Interpersonal Behaviours among Nurse Educators in the Workplace

This subtheme addressed role recognition, conflicts between nursing service and teaching, and the complexity of student evaluation.

*“Novices discussed being in a familiar hospital provided an extra layer of transitional comfort to the position. Although this was also acknowledged by full-time faculty, there was concern of role conflict between the nursing service and teaching roles, and this could occur if placed on the same unit, potentially causing “a conflict of interest” and affecting clinical staff interaction.”* (33)

*“Complexity of evaluation emerged as a major category by the experienced full-time members. “It*’*s one of the hardest things we do.” Many verbalized that the adjuncts were not taught how to give student feedback, were not confident about the evaluation process, feared the student appeal process, and desired to be liked and keep the student happy. This coincided with the category of role conflict between teacher and student friend*” (33)

“*I think there is a tension…that is a by-product of us trying. It is that continual need to be sure that our pedagogies engage this generation, whatever this generation*’*s quirks are.*” (38)

#### Subtheme 1.4: Nurse Educators’ Unattractive External Environment

The nurse educators’ external environment presented unattractive elements, such as narratives of subordination, the toil of the journey, expectations for women in the academy, and challenges related to nursing curricula and syllabi.

*“I can lose a day answering emails, several times a week. Because the students have the expectation that you will respond to them quickly, and the university just spews out emails all the time.”* (39)

*“All of the participants recognized that a career in academia required them “to work long hours” and “out of hours”; ultimately causing each academic to ask themselves ‘how hard do I want to work?’ Concerns about workload were illustrated, in part, by the attrition of two of the participants leaving shortly after the first set of interviews. Their thoughts had become clarified as result of the “all-consuming administration” and decided to take up other job opportunities that gave more time to pursue their passion in clinical research.”* (36)

### Theme 2: Life integration of nurse educators

This theme focused on the nurse educators’ personal and family life aspects. It considered the alignment of parenthood and academia, the influence of environmental comfort, and the emotions and feelings nurse educators experienced in their academic careers.

#### Subtheme 2.1: The Attractive Alignment of Parenthood and Academia

The alignment of parenthood and academia was considered attractive, with environmental comfort acting as a protective element. Nurse educators found comfort in familiar hospital settings and relied on clear boundaries to manage their workload.

*“I had young children…the long hours working in the hospital and all the different shifts, the holidays were gone”* (34)

“*Novices who felt comfortable calling and communicating with the facilitator or other full-time faculty had a more positive experience*” (33)

#### Subtheme 2.2: The Ultimate Costs of Downplaying Parenthood in Academia

The cost of parenthood in academia was evident, with participants feeling the need to “pass” as the ideal worker by downplaying their parenthood status. The world of academics and family life often merged, requiring a delicate balance from nurse educators

*“At work, as long as I was not talking about my children or associated with an item to remind others of my motherhood status (e.g., stroller), I could “pass” as the ideal worker”* (40)

The study's findings reflected nurse educators’ complexities and challenges in integrating their work and personal lives. The thematic analysis revealed positive and negative aspects of work-life integration, providing valuable insights for addressing these challenges and enhancing this population's well-being.

## Discussion

The intersection of parenthood and academia among nurse educators is rich in rewards and challenges. Montero-Diaz (40) eloquently delineates the myriad advantages of the academic realm, including flexible work hours, autonomous time management, and the opportunity to pursue profound passions within one's career trajectory. Moreover, insights from Guy and Arthur (41) underscore the profound significance of motherhood within academia, revealing that despite encountering formidable obstacles, mothers in this sphere cherish the invaluable moments spent with their children. Wilton and Ross (42) further corroborate these sentiments, emphasising the quest for fulfilling workplace experiences that complement motherhood's multifaceted roles.

Nevertheless, alongside these attractive facets lie profound challenges for female nurse educators. The pervasive influence of motherhood on women's academic trajectories is evident (43), particularly for those in the throes of balancing professional pursuits with childcare demands. Indeed, the landscape appears daunting for working mothers, with the prospects varying significantly depending on geographical location and institutional support systems (44). Confronted with restrictive structures and enduring stereotypes, working mothers contend with pervasive societal judgments that permeate academic spheres (45).

Inequities entrenched within higher education institutions exacerbate these challenges, perpetuating what is colloquially termed “motherhood penalties” (46). Navigating multiple roles places mothers at a distinct disadvantage, amplifying the strain of reconciling workplace demands with the rigours of academic life (43). Wilton and Ross (42) echo these sentiments, highlighting the sacrifices made by both men and women in academia to strike a delicate equilibrium between professional commitments and familial responsibilities.

Keefe (47) advocates for a bimodal approach among nurse educators that prioritizes a holistic legacy encompassing the traditions of higher education—spanning research, teaching, and community engagement. This paradigmatic shift underscores the imperative of nurturing a balanced, healthy work-life integration, resonating with broader societal aspirations. Similarly, Weinstein (48) underscores the indispensable need for self-care amidst the intricacies of professional and personal domains, advocating for more outstanding agency in navigating the delicate interplay between these spheres.

The discourse surrounding work-life integration among nurse educators illuminates the complex interplay between personal fulfillment, professional aspirations, and societal expectations. As the field continues to evolve, fostering an inclusive environment that prioritizes holistic well-being emerges as an imperative, underscoring the need for concerted efforts to recalibrate institutional structures and societal norms.

### Strengths and limitations

Seven studies were retrieved for this meta-synthesis, and their credibility was confirmed using STARLITE. Most of the studies were compliant with the criteria indicated on the STARLITE. The findings from the seven studies concur regarding nurse educators’ challenges and experiences of work-life integration. No studies were conducted in Africa on this topic, calling for research of this nature to be conducted in this region. Another limitation is that only English articles were included in the review. Most of the articles were published in nursing education journals or nursing practice journals, which may have led to an omission of possible valuable information outside the abovementioned journals.

## Conclusion

Nurse educators navigate a complex landscape where work demands often clash with personal aspirations. Despite the pervasive challenges in balancing these spheres, nurse educators tend to internalise their struggles as personal failings rather than acknowledging systemic barriers (49). This self-perception not only exacerbates the strain on their mental well-being but also underscores the urgent need for supportive work environments conducive to harmonising professional responsibilities with personal life. Trepal and Stinchfield (50) advocate for individual accountability in delineating boundaries between work and personal domains, yet the onus cannot solely rest on the shoulders of educators. Academic institutions must proactively enhance workplace policies, including provisions for improved maternity leave, accessible childcare options, and robust social support systems. Such initiatives are pivotal in alleviating nurse educators’ burdens and fostering an environment where work-life integration thrives. Effective leadership from line managers is paramount. By demonstrating empathy and offering encouragement, managers can cultivate a culture of understanding that empowers nurse educators to confront and navigate personal challenges with resilience.

In essence, this study serves as a clarion call to action. It implores academic institutions and employers to recognise the profound impact of work-life integration on the well-being of nurse educators. By instituting supportive policies and nurturing empathetic leadership, we can pave the way for a future where nurse educators flourish both personally and professionally.

## Data Availability

The original contributions presented in the study are included in the article/Supplementary Material, further inquiries can be directed to the corresponding author.
